# 

**Published:** 1863-02

**Authors:** 


### BOOKS RECEIVED.

*The Principles and Practice of Obstetrics. By* GUNNING S. BEDFORD, M. D., *Professor of Obstetrics, the Diseases of Women and Children, and Clinical Obstetrics, in the University of New York; author of "Clinical Lectures on the Diseases of Women and Children." Illustrated by four Colored Lithographic Plates and ninety-nine wood engravings. Third Edition, carefully revised and enlarged. New York:* WILLIAM WOOD & CO., 61 *Walker street.*

*The Physiology of Reproduction, and Origin of Sex; giving a full and Scientific explanation of the new discovery by which either sex may be procreated at the option of the parents, and being the only correct exposition of the subject ever published. By* J. H. SANBORN, M. D. *New York:* OLIVER TRAMWELL & CO., 1863.

*Biennial Report of the Board of Trustees of the Michigan Asylum for the Insane, for the years* 1861–2, *By Authority. Lansing:* JOHN A. KERR & CO.

*Reporter of the New Patent Artificial Leg, published by* D. DEFORREST DOUGLASS, *Inventor and Manufacturer, Springfield, Mass. Fourth Edition,* 1863.

*The Surgical Adjuvant, and reporter of Surgical Apparatus, Artificial Limbs, etc., by* E. D. Hudson, M. D. *New York: Office, Clinton Hall, up stairs.*

*On the Pathological Basis of the Treatment of Joint Diseases. By* Henry G. Davis, M. D. *Read before the New York Academy of Medicine.*

*Sanitary Commission, No.* 39. *Third Report concerning the Aid and Comfort given by the Sanitary Commission to Sick Soldiers passing through Washington. By* Frederick N. Knapp, *Special Relief Agent.*

*Sanitary Commission, No.* 59. *Fourth Report concerning the Aid and Comfort given by the Sanitary Commission to Sick Soldiers passing through Washington. By* Frederick N. Knapp. *Special Relief Agent.*

*Bulletin of the New York Academy of Medicine. Nos.* 1–4. *Subjects: Albuminuria—Vomiting in Pregnancy. New York:* Bailliere Brothers, 440 *Broadway.*

Subscriptions received for the current year of the Bulletin. Price, $1.

